# An Early Underwater Artificial Vision Model in Ocean Investigations *via* Independent Component Analysis

**DOI:** 10.3390/s130709104

**Published:** 2013-07-16

**Authors:** Rui Nian, Fang Liu, Bo He

**Affiliations:** School of Information Science and Engineering, Ocean University of China, 238 Songling Road, Qingdao 266100, China; E-Mails: nianrui_80@163.com (R.N.); lfpkq@126.com (F.L.)

**Keywords:** ocean investigations, AUV, early human vision system, ICA, underwater vision model

## Abstract

Underwater vision is one of the dominant senses and has shown great prospects in ocean investigations. In this paper, a hierarchical Independent Component Analysis (ICA) framework has been established to explore and understand the functional roles of the higher order statistical structures towards the visual stimulus in the underwater artificial vision system. The model is inspired by characteristics such as the modality, the redundancy reduction, the sparseness and the independence in the early human vision system, which seems to respectively capture the Gabor-like basis functions, the shape contours or the complicated textures in the multiple layer implementations. The simulation results have shown good performance in the effectiveness and the consistence of the approach proposed for the underwater images collected by autonomous underwater vehicles (AUVs).

## 1. Introduction

The 21st century is the Era of the “Ocean”. There has been a growing trend towards the deployment of the ocean blueprint all over the World. Ocean investigations, an emerging direction to explore the underwater environment conditions and changes, discover the inherent nature of the movement, behaviors and activities for marine life, and describe the physical, chemical, and biological factors on the sea bed and ocean surface, have shown great prospects in oceanography, fisheries, geophysics, biology, and other marine related surveys and applications [1–4].

Vision is one of the dominant senses in ocean investigations. Traditional studies mainly focus on the on-site observations periodically conducted by marine scientists, which is not only time consuming, requiring human concentration, but also make it difficult to obtain a global analysis. Consequently, the use of underwater vision systems, either tethered to a vessel or shore-based facility, or deployed by remotely operated vehicles (ROVs) and autonomous underwater vehicles (AUVs), has increased rapidly over the last decade [5–9].

Humans possess such good visual capabilities since childhood. In the human vision system, visual information passes through the various processing layers along the visual pathway, such as the retina, the lateral geniculate nucleus (LGN), the primary visual cortex (V1), the prestriate cortex (V2), and beyond [10]. With a simple glance, humans are easily able to remember and judge specific objects despite phenomena like occlusion, illumination problems, deformations or viewpoint changes. However, it is difficult to develop the fascinating human visual abilities in a computer vision system, due to the following obstacles: (1) all sorts of occurrences of variation coming from optical, spatial, and temporal factors; (2) the intelligent visual strategies and procedures that can exhibit and simulate human-like behaviors and performances. Hereby, one central topic for ocean investigations is to establish and facilitate a human visual simulation mechanism appropriate for understanding the underwater environment, on a basis of a proper intelligent vision system that can reasonably capture the characteristics of underwater objects from diverse perspectives.

Dating back to the first approaches to human vision simulation models, one of the popular theories about the functional role of visual perception, the efficient coding theory, hypothesizes that the early visual perception processing serves to capture the statistical structure of the visual stimulus by removing the redundancy in the visual outputs [11]. Therefore, some linear implementations of efficient encoding, such as the Independent Component Analysis (ICA) [12], have been used to explain the very first layers of the visual information processing system in the cerebral cortex and learn visual features exhibiting the receptive field properties of V1 simple cells [12,13].

The general framework of ICA was originated in the early 1980s as a seminal work for blind source separation studies [14–17]. Jutten and Hérault first proposed an adaptive algorithm in a simple feedback architecture based on a neuromimetic approach to solve such a problem [14,15]. Furthermore, Comon introduced the concept of ICA and put forward the cost functions of the mutual information minimization between the sensors [16]. In parallel, unsupervised learning rules have been proposed to maximize the mutual information based on information theory by Linsker, Becker and Hinton, *etc.*, closely related to the redundancy reduction principle for the efficient encoding in the human visual neurons [11,18]. The hypothesis that each neuron should encode features that are statistically independent from others has been explored for the early visual processing by Attik. Nadal and Parga showed that in the low-noise case, the maximum of the mutual information between the input and output of a neural processor implied that the output distribution was factorial. Roth and Baram, Bell and Sejnowski independently derived stochastic gradient learning rules [19,20]. Their adaptive methods are more plausible from a neural processing perspective than the cumulant-based cost functions. Amari proposed to replace the steepest descent gradient algorithm by the natural gradient, and provided the better calculation and convergence coefficient recursion formula in the orthogonal coordinate [21,22]. Hyvarinen put forward the fast fixed point independent component analysis algorithm by a statistical analysis of the maximum nongaussianity framework that has shown excellent performance with less calculation and speedy convergence [23–25]. Shan developed the Recursive ICA (RICA) to capture nonlinear statistical structures of the visual inputs for the natural images since there is in fact still significant statistical dependency between the variance of the ICA outputs [26], and then carried out recognition tasks by the sparse coding learned from the natural images [27,28]. Several extensions of the linear ICA algorithm have been proposed to reduce such residual nonlinear redundancy, with an explicit or implicit aim of explaining higher perceptual layers [29–32].

In this paper, we try to further explore the functional roles of the early underwater artificial vision model by means of the multi-layer ICA framework. The rest of the paper is organized as follows: in Section 2, the general ocean investigations will be briefly introduced, including the established computer vision systems, the underwater imaging models and a novel image enhancement approach for our work. In Section 3, the basics of the early human vision system and the ICA algorithm will be outlined. In Section 4, our underwater artificial vision model with the hierarchical ICA architecture is developed in detail. In Section 5, the simulation is given in support of the developed scheme. Section 6 presents the conclusions.

## 2. Human Vision System

### 2.1. Basic Vision Structure

Vision plays one of the most important roles and constitutes the basic sensory capability in the brain functionality [10]. Vision begins with an organ that is sensitive to light. At the front of the eye is a transparent refracting surface (the cornea) and at the back lies a layer of neural tissue (the retina) with light-sensitive elements (the photoreceptors). In between, there is a variable aperture (the pupil) and behind that a second refracting element (the lens). The lens affords the possibility of changing the focal length or power (accommodation) to adjust for the distance to an object of interest.

The eye's output fibers that form the optic nerve eventually project to a relay nucleus in the brain where the different types of visual information are passed along for their initial processing in the primary visual cortex (V1). Numerous properties of the visual image are transmitted separately, and in parallel, to the primary visual cortex where certain basic features are now extracted. As the information leaves the primary visual cortex, this parallel organization of visual information is maintained, but now each type of information is sent to a different, largely independent higher cortical center. Thus, the visual system at this level has not only a parallel but also a modular organization.

Conscious perception of a visual scene with all of the various properties reintegrated occurs later at higher levels of the visual system. At this stage, the various components of the scene, each with their attendant characteristics, are recognized and understood. A complete interpretation of the visual world involves not only identifying different objects and their spatial relationship to each other but also their significance. This demonstrates that visual perception is, by and large, not hard-wired and illustrates the inherent flexibility of the visual system. Recognition of the object of interest occurs in areas of the inferotemporal cortex (PIT and AIT). A motor response originates in the prefrontal cortex (PFC) and is then transmitted via the premotor cortex (PMC) to the primary motor cortex (MC), which produces the response in skeletal muscles via the spinal cord.

### 2.2. Typical Vision Characteristics

In the human vision system, when the visual stimulus travel from the retina in the eye to the lateral geniculate nucleus (LGN) and then via the primary visual cortex (V1) on to higher visual centers (V2, V4, *etc.*), there are a number of typical characteristics among the visual perception processing layers:

#### Modality

Different parts of the brain share similar anatomical structures and it is likely that they are also working under similar computational principles. For example, fMRI studies have shown that removal of one sensory modality leads to neural reorganization of the remaining modalities [33], suggesting that the same principles must be at work across modalities. One of the intriguing consequences of this modular arrangement is that, if a person suffers localized damage to the brain, involving one of the higher visual centers, the deficit can be very specific in nature.

#### Redundancy Reduction

It has long been found that the functional role of some visual perception processing serves to capture the statistical structure of the visual stimulus so that appropriate action decisions can be made to maximize the chance of survival. Barlow provided the insight that the statistical structure is measured by the redundancy of the stimuli and that completely independent stimuli cannot be distinguished from random noise. One possible way for the neural system to describe the statistical structure is to remove the redundancy in the visual outputs, which refers to the efficient coding theory or the redundancy reduction principle.

#### Sparseness

The neurons in V1 take each input from a number of geniculate neurons, and any individual neuron can only see a small portion of the image that the eyes are viewing. This small region is the receptive field and can be characterized as being localized, oriented, and bandpass [34]. Olshausen and Field [35,36] have indicated that the neural networks in the human vision system could perform sparse coding of the learnt features qualitatively similar to the receptive fields of simple cells in V1, which searches the succinct representations of the visual stimulus.

#### Independence

Given only the unlabeled input properties of the visual image with virtually no prior knowledge on the signals, the neurons in V1 can learn certain basic features that capture the independent higher-level features in parallel. The basis functions can be learned to resemble the receptive fields of the neurons in the visual cortex and model the basic cortical processing of visual and auditory information with the linear efficient encoding [35–37].

### 2.3. The Basics of ICA

ICA was initially proposed to solve the blind source separation (BSS) problem, *i.e.*, given only the sensor observations that are unknown mixtures of a set of underlying sources, the task is to separate the mixed signals and recover the original independent sources [14–17]. Neither the mixing process nor the distribution of sources is known in advance during the process. ICA algorithms have been further applied to explain the functional roles of the first stage of cortical visual processing, *i.e.*, the V1 simple cells. The redundancy reduction principle forms the foundation of ICA algorithms.

The basic ICA model can be denoted as a linear superposition of the basis functions in a Bayesian framework [38]:

(1) x=As+ε

where the observed input vector **x** = [*x*_1_,…,*x_i_*,…,*x_d_*]′ ∈ R*^d^* represents the image patch in a *d*-dimensional space, **s** = [*s*_1_,…, *s_i_*,…,*s_q_*]′ ∈ *R^q^* align corresponds to the independent feature representation of the original image patch **x** in a *q*-dimensional space, *ε* is additive Gaussian noise, the input image patch **x** is assumed to be a linear mixture of underlying signals **s** with the additive Gaussian noise *ε*, and **A** refers to a *d* × *q* matrix of the basis functions denoting the dictionary of elementary features that generate the observations **x**. Generally speaking, there are two assumptions imposed on the underlying features **s** in the ICA algorithms. First, the underlying features are statistically independent, *i.e.*, *p*(**s**) = Π*_i_ p*(*s_i_*), which integrates the efficient encoding theory into the ICA. Second, the marginal distribution of the features *p*(*s_i_*) follow the sparse distributions, *i.e.*, a specific low entropy code where the probability distribution of each feature's activity is unimodal and peaked around zero. The sparseness is desirable because: (1) it allows the neural system to easily assign actions to the corresponding inputs, (2) it save metabolic cost, (3) many real-world signals do follow the sparse distribution [39]. Therefore the goal of the ICA is to find the basis function matrix **A** so that the underlying feature values s can be statistically independent over an ensemble of the images and bear the sparse structure.

In the implementation, both the objective function and the optimization algorithm will play the key roles in the ICA generative model. The statistical property of the ICA such as the robustness and the consistency depends much on the choice of the objective function, and the nature of the ICA such as the convergence speed and stability depends a lot on the optimization algorithm. A general formulation for the ICA criterion is based on the concept of the mutual information:

(2) $$\begin{array}{l} I[\text{s}]=J(\text{s})‐\sum_{i}J(s_{i})\\ J(\text{s})=KL[p(\text{s}),p_{g}(\text{s})]=H_{g}(\text{s})‐H(\text{s}),H(\text{s})=‐\int p(\text{s})\log p(\text{s})ds \end{array}$$

where *I* refers to the mutual information among the independent component **s** = [*s*_1_,…,*s_i_*,…,*s_q_*], *p*(**s**) is the probability density function of **s**, *p_g_*(**s**) corresponds to the Gaussian distribution with the same covariance matrix as **s**, *H* and *H_g_*(**s**) are respectively the differential entropy of **s** and the Gaussian random variable, *J* is the negentropy with the *KL* divergence that is normalized to have the appealing invariant property for the linear transformations. Here to minimize the mutual information *I* of the transformed components **s** is in fact the maximization of the negentropy *J*. The farther away from the Gaussian distribution the independent component **s** is, the less the mutual information *I* is, and the more the negentropy *J* is. The objective function of the ICA is hereby to maximize the negentropy.

## 3. Ocean Investigations

### 3.1. Underwater Vision Systems

In the ocean investigations, underwater observations can provide real time information about the specific underwater environments. At the same time, they are relatively benign, resulting in limited damage of the habitat, making them particularly suitable for monitoring sensitive objects and areas or threatened and endangered species.

The establishment of the underwater information collection over time depends on a vision system providing insight of the ocean investigations with great reliability, accuracy and the cost reduction, which highlights a need to balancing the video quality and the physical limitations (range, resolution, frame rate and compression) while maximizing the memory capacity and battery power. Recently, the autonomous and remote vision system has become a predominant tool due to the continuous navigation and sampling it offers to achieve simultaneous observations over large areas.

**Figure 1.**
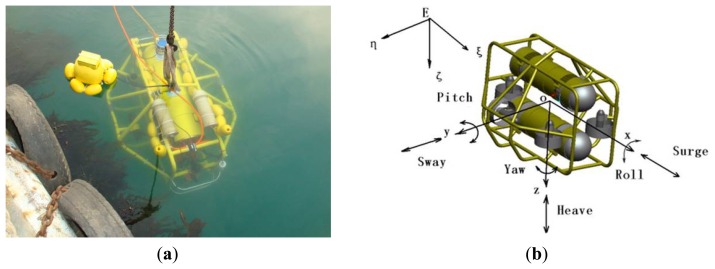
(**a**) C-Ranger is in deployment. (**b**) The coordinate system of C-Ranger platform.

In this context, the C-Ranger AUV system with a number of sensors on board is used here as the basis of our underwater vision system. The C-Ranger is an open-frame AUV with the sizes of 1.6 m × 1.3 m × 1.1 m (length, width and height), as shown in Figure 1.

The AUV has good maneuverability due to its five DOFs, including surge, heave, roll, pitch, and yaw. The thrust system of this platform consists of five propeller thrusters, where two thrusters paralleling to the bow direction are installed on the abdomen to provide horizontal thrust for mainly controlling the surge and yaw, while the other three thrusters are employed to provide vertical thrust to control the heave, roll, and pitch, two of which are installed on both sides of the bow, and the remaining one is installed on the rear of the vehicle. The upper hull of the C-Ranger is the instrument compartment housing sensors, two industrial computers, communication module, internal monitoring module and other equipment, while the lower hull is the power and thrust system composed of lithium-ion batteries, power management module, motor-driver module, *etc.* The maximum speed of the C-Ranger is 3 knots, and it can operate for up to 8 h when fully charged (tested at a speed of one knot). The C-Ranger AUV is designed for neutral buoyancy and the maximum depth is 300 m below the sea surface. The sensors installed on the C-Ranger AUV can be basically divided into two groups: the internal and the external. Internal sensors include digital compass, gyro, attitude and heading reference system (AHRS) and pressure sensor. External sensors include mechanical scanning sonar, Doppler Velocity Log (DVL), altimeter, CCD camera and GPS. An external video monitoring device with high resolution and sensitivity, the Kongsberg Maritime OE14-376 Light Ring Color Camera, has been installed in the C-Ranger AUV for the composite recording missions, providing a 43.5° diagonal angle of view in water and supporting a water depth of 3,000 m.

### 3.2. Underwater Imaging Model

Underwater images are essentially characterized by poor visibility, which result from the specific properties of light in water, such as the limited range, non uniform lighting, low contrast, diminished colors, blur imaging and so on. Moreover, owing to the complexity of the marine environment, the optical properties can often be modified, so the underwater images may present large temporal and spatial variations. The Jaffe-McGlamery model is well-known in the analysis of the underwater image formation [40–42], with the following basic assumptions:

#### Linear Superposition of Irradiance

The underwater imaging propagation process at a specific point can be decomposed into three additive linear components:

(3) $$E_{t}=E_{d}+E_{f}+E_{b}$$

where *E_t_*, *E_d_*, *E_f_*, *E_b_* are respectively the total irradiance, the direct component, the forward-scattered component and the backscattering. The direct component is the light reflected by the object surface and entered the camera without scattering. The forward scattering is the amount of the randomly deviated light reflected by the object on its way to the camera, which enters the camera after scattered at a small angle and causes blurring of the image features. The backscattering is a significant fraction of the light reflected not by the object but still entered the camera due to the suspended particles in transmission, which causes undesirable differences of contrast and masks the details of the scene, though visibility may indeed be augmented with artificial lighting.

#### Attenuation Modeling for Medium Light Interaction

The light intensity in the Jaffe-McGlamery model is an exponential decay with distance:

(4) $$L_{i}(d)=L_{0,i}\exp(-c_{i}d)$$

where *i* is the wavelength of light, *d* is the distance traveling in a liquid, *L_i_*(*d*) is the light intensity of wavelength *i*, *L*_0,_*_i_* is the light intensity of wavelength *i* at the light source, and *c_i_* is the attenuation coefficient at wavelength *i*, respectively. The attenuation usually leads to a hazy and poorly contrasted image background.

Besides, the Macroscopic floating particles (marine snow), can also be considered as unwanted signals. When considering the magnitude, backscattering and marine snow are the greatest degradation factors, forward scattering comes second and the attenuation follows closely.

#### 3.3. Image Quality Enhancement

Due to a great many impacts on the underwater image quality mentioned above, image enhancement is one of the key issues to optimize our understanding. In this paper, we present a generic parameter-free enhancement method to make a total abstraction of the image formation process, reduce underwater perturbations, and correct the contrast disparities caused by the attenuation and backscattering, without the prior knowledge of the depth, the distance and the water quality.

The color space model of the image is first converted into the YCbCr space to concentrate only on the luminance channel which corresponds to the intensity component. The homomorphic filtering is adopted to correct non uniform illumination, enhance contrasts and sharpen the edges at the same time. Wavelet decomposition is further introduced to the homomorphic filtering for image denoising. The wavelet base is nearly symmetric orthogonal with a bivariate shrinkage exploiting interscale dependency.

The underwater image is first represented as the product of the illumination and the reflectance:

(5) I(x,y)=i(x,y)r(x,y)

where *I*(*x*,*y*) is the collected image, *i*(*x*,*y*) is the illumination multiplicative factor, and *r*(*x*,*y*) is the reflectance function. When taking the logarithm of the image:

(6) z(x,y)=lnI(x,y)=lni(x,y)+lnr(x,y)

the Fourier transform of the log-image becomes:

(7) $$Z(u,v)=F_{i}(u,v)+F_{r}(u,v)$$

where *F_i_*(*u*,*v*), *F_r_*(*u*,*v*) are respectively the Fourier transform of ln *i*(*x*,*y*) and ln *r*(*x*,*y*).

One kind of high-pass filter *H* (*u*,*v*) that merges the property of wavelet decomposition is introduced here to decrease the contribution of low frequencies and amplifies the contribution of mid and high frequencies, sharpening the object edge in the image:

(8) $$\begin{array}{c} S(u,v)=Z(u,v)H(u,v)=F_{i}(u,v)H(u,v)+F_{r}(u,v)H(u,v)\\ H(u,v)=H(j,\omega_{h},\omega_{l})=(r_{h}-r_{l})\frac{1}{1+{\left(\frac{k_{c}}{c\sqrt{\omega_{h}^{2}+\omega_{l}^{2}}\left(2^{j}\right)}\right)}^{2n}}+r_{l} \end{array}$$

where *j* is the level of the wavelet decomposition, *k_c_* is the stopping coefficient, *ω_h_* and *ω_t_* are respectively the horizontal and vertical weights, *c* is a constant between the two parameters *r_h_* and *r_l_*, which is introduced to control the filter function sharpening. The inverse transform is then taken to come back to the spatial domain:

(9) $$s(x,y)=i^{\prime \prime }(x,y)+r^{\prime \prime }(x,y)$$

Taking the exponent to *s*(*x*, *y*) will obtain the filtered image *I*'(*x*,*y*):

(10) $$I^{\prime }(x,y)=e^{s(x,y)}=e^{i^{\prime \prime }(x,y)}e^{r^{\prime \prime }(x,y)}=i^{\prime }(x,y)r^{\prime }(x,y)$$

where *i*'(*x*,*y*), *r*'(*x*,*y*) are respectively the illumination and the reflectance.

## 4. Early Artificial Vision Model

### 4.1. General Vision Model

The idea of the early artificial vision model here is that we try to apply one hierarchical ICA architecture into the underwater images for ocean investigations so that the existing residual nonlinear dependency problem can be modeled and transformed into an easier modular solution with multiple layers. Since the linear ICA algorithms have been so successful in explaining the very first layer of perceptual information processing in the cerebral cortex, it seems reasonable to hypothesize that the higher layers might also be explained by a linear ICA model.

**Figure 2.**
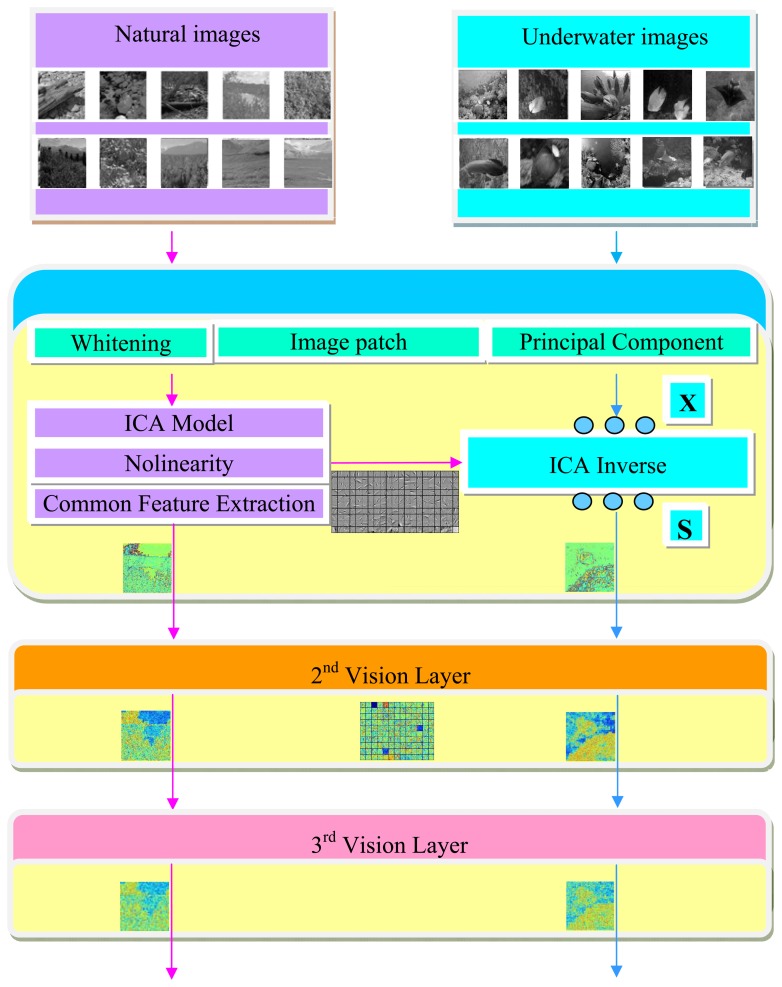
The flow chart of our approach.

The common feature hypothesis will be first taken to extract the basis function in common form of the universal images patches for the underwater vision model. Some sensory input constraints are imposed and the recursive application will be derived by the generative model of ICA in each layer. The flow chart of our approach is shown in Figure 2, where the left part represents the learning process of the important parameters extracted from the universal images, and the right part is referred as the test process for the underwater images. Before formally simulating the basis function from the universal images, some preprocessing has been first done, such as dividing the images into patches, whitening, and the dimensionality reduction. The information that provides no interesting structure will be discarded and the activation function will be taken to make sure that the marginal distribution obeys the input requirements for the next layer. A number of common visual features can then be extracted from randomly collected universal images, the embedding basis function in each layer will be further adopted for the underwater images.

### 4.2. Common Feature Hypothesis

In the human vision system, it is found that the low-level visual layers, such as retina, LGN and V1, are shared components that process all the visual information we perceive. These layers develop and mature gradually since childhood, and provide the basis with common features from the scenes encountered for all the visual tasks in life.

Therefore, the concept of the common feature hypothesis suggests that all visual stimuli share characteristics in common such that the knowledge from one set of visual stimuli can be applied to a completely different one. So here we try to extract those common visual features which is essential for underwater vision from a set of the universal images, e.g., the natural images, and provide the information for ICA in the next step. Let there be *Q* images taken under the sea, $\left\{I_{1}^{S},\cdots ,I_{i}^{S},\cdots ,I_{Q}^{S}\right\}$, where $I_{i}^{S}$ denotes the *i* th underwater image. Suppose that the number of the natural images is *N*, $\left\{I_{1}^{U},\cdots ,I_{i}^{U},\cdots ,I_{N}^{U}\right\}$, there must be some inherent common visual features **A** that can be extracted both in the natural images and the underwater images:

(11) $$F_{\text{nature}}\subseteq f(I_{1}^{U},\cdots ,I_{i}^{U},\cdots ,I_{N}^{U}),F_{\text{sea}}\subseteq f(I_{1}^{S},\cdots ,I_{i}^{S},\cdots ,I_{Q}^{S}),\text{A}\in \{F_{\text{nature}}\cap F_{\text{sea}}\}$$

where *f* denotes the attribution extraction function, *F_nature_* and *F_sea_* are respectively the typical features obtained from the output of the function *f* by the natural images and underwater images, and **A** represents those knowledge that are shared by the different sources of the visual stimuli.

### 4.3. Artificial Vision Model with ICA

#### Pre-whitening

**Figure 3.**
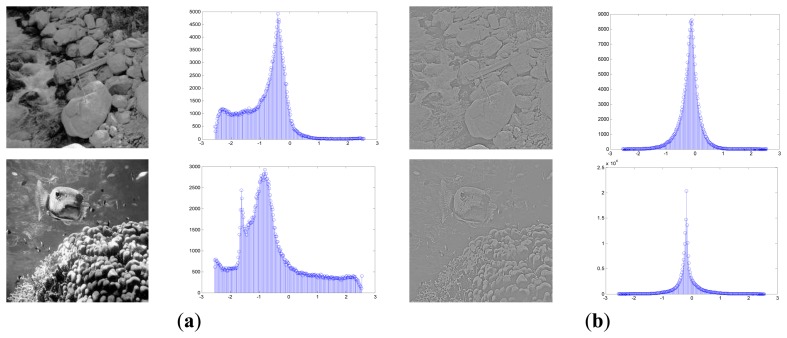
The distribution of the pixel values before and after pre-whitening transformation. (**a**) Original image and distribution. (**b**) Pre-whitened image and distribution.

Suppose the size of each input image is *M* × *M*, the images are first transformed by a pre-whitening filter and then normalized to follow a Gaussian function with the zero mean vector and the unit variance [36]. It is believed that a surprising fact in the human vision system is that there exists the marginal distribution regularization process and the sensory inputs are whitened in the retina and the LGN before the transmission to V1 [12,18,23]. The performance of the early artificial vision model depends much on the form of the input data. If the observed data strongly deviate from our assumption, the results could be errant no matter how much effort we put into the model parameter estimation. Besides the functional role of removing the second-order pairwise redundancy as the natural images obey the 1/*f* power law in the frequency domain [43], pre-whitening might also serve as formatting the sensory input for the cortex so that the basis function could cover a broad range of spatial frequencies. The steps of the pre-whitening process are as follows. To avoid the boundary effects, before dividing the natural images into all the possible image patches, we will first cut a number of pixels *m* off the boundary and change the *i* th input image into a *D*-dimensional vector *I_D_* with *D* = (*M*−*2m*) × (*M*−*2m*). Afterwards, we divide each input image *I_D_* into all the possible image patches in a range of *d* = *p* × *p* size. The sample set **X** with *n* = *N* × *D*/*d* image patches then takes on a *d* × *n* matrix, **X** = (***x***_1_,…***x****_i_*, …,***x****_n_*). Figure 3 shows examples of the pre-whitening process, which regulates the marginal distribution of the original images to follow a generalized-Gaussian-like distribution.

#### Whitening

We first convert **X** to the centered matrix **X̅** by substracting the mean vector matrix of **X**. The covariance matrix of **X̅** can then be calculated and represented as **C** = **X̅X̅**^T^ = **UΛU**^T^, where **Λ** = diag[*λ*_1_,…*λ_i_*,…*λ_d_*] refers to the diagonal matrix of the eigenvalues, **U** = [***u***_1_,…,***u****_i_*,…,***u****_k_*] denotes the eigenmatrix composed of the eigenvectors. After adjusting **Λ** in a descending order and arranging the corresponding eigenvectors, the Principal Component Analysis (PCA) approach is adopted here to select the first *q th* eigenvectors and form the whitening matrix **V** in a *q* × *d* size with $\text{V}=\Lambda^{-\frac{1}{2}}{\text{U}}^{\text{T}}$, where **Λ** turns to be a *q* × *q* matrix and **U** a *q* × *d* matrix. The centered sample matrix **X̅** will then be further whitened by **Z** = **VX̅**, where the whitened sample matrix **Z** is in a *q* × *n* size. After whitening, all the components of the whitened matrix **Z** are uncorrelated to each other and the variance of each component turns into 1, which is more convenient to the ICA processing.

#### Rising dimensionality

After the ICA operation with **Z** = **AS**, we get the independent component representation **S** in a q × n size. In order to reconstruct the original images, the dimensionality of **S** need to be the same as it is in the sample set **X** so here the dimensionality of **S** is further increased into a *d* × *n* size by whitening again with **S** = **V**^T^**S**. The whitened independent component representation **S** is also fit for the requirements of the inputs for the multiple layer ICA architecture.

#### Nonlinearity

A further development to convert the direct ICA output **S** into a standard distribution for the input of the next layer will be derived here in a nonlinear way. In fact, the outputs of those classical ICA algorithms which resemble the receptive fields of the simple visual cells on the natural images typically follow the symmetric and sparse marginal prior [12,23,27]. Motivated by that, the ICA filter response can be well approximated by a generalized Gaussian distribution.

Assuming that the direct ICA output **S** obeys the following probability density function (pdf):

(12) $$f(\text{s};\sigma ,\theta )=\frac{\theta }{2\sigma \Gamma (1/\theta )}\exp\{-\frac{{\left|\text{s}\right|}^{\theta }}{\sigma }\}$$

where *σ* > 0 is a scale parameter and *θ* > 0 is a shape parameter and Γ denotes the gamma function, $\Gamma (x)=\int_{0}^{+\infty }t^{x-1}e^{-t}dt$ [44]. The overall nonlinear transformation function will then be:

(13) $$g(\left|\text{s}\right|)=F^{-1}(\gamma \text{(}\frac{\left|{\text{s}}^{\theta }\right|}{\sigma^{\theta }},\frac{1}{\theta })/\Gamma (\frac{1}{\theta }))$$

where *g* is the coordinate-wise nonlinear activation function that formats the marginal distribution to Gaussian, *F* represents the cumulative density function of the standard normal distribution and γ is the incomplete gamma function, $\gamma \text{(x},\text{y)}=\int_{y}^{+\infty }t^{x-1}c^{-t}dt$. Here three consecutive steps are involved to transform the ICA output **S** into such a normally distributed random variable **v** : (1) Discard the signs of the ICA feature that provide no interesting structure in the vision model. It has been argued that the signs of the ICA filter outputs do not carry any redundancy among the dimensions and some algorithms have implicitly or explicitly discarded the signs [29-32]. We take place of the ICA filter output **S** by the new random variable **v** so that **v** could bear the following pdf:

(14) $$g(\text{v};\sigma ,\theta )=\frac{\theta }{\sigma \Gamma (1/\theta )}\exp\{-\frac{{\text{v}}^{\theta }}{\sigma }\},0\leq \text{v}\leq \infty$$

(2) Transform **v** to a uniform distribution by its own fitted cumulative distribution function (cdf), $\text{v}=\gamma \text{(}\frac{{\text{v}}^{\theta }}{\sigma^{\theta }},\frac{1}{\theta }\text{)}/\Gamma (\frac{1}{\theta })$, 0 ≤ **v** ≤ ∞. For each dimension *s_j_* of **S**, the empirical cdf of the absolute value |*s_j_*| will be first estimated by calculating the histogram of |*s_j_*| in the range of the bins between b_min_ and b_max_ and the size of each bin is b. (3) Transform **v** to a Gaussian distribution by applying the inverse cdf, **v** = *F*^−1^(**v**).

In this way, the activation function discards the signs of the ICA filter outputs and converts the marginal distributions to the Gaussian distributions, so that the feature extracted from the previous layer can further feed into the next layer as the new inputs.

#### Hierarchical architecture

**Figure 4.**
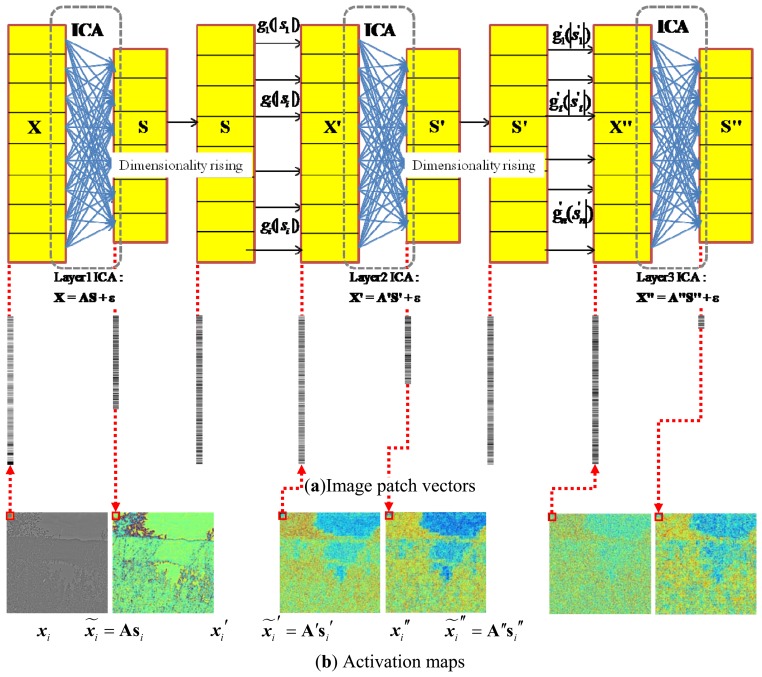
The hierarchical ICA architecture. (**a**) Image patch vectors; (**b**) Activation maps.

A collection of the individual ICA filter units will be organized into a hierarchical model. The model is motivated by the idea that higher layers of the visual pathway, such as V2, might work under similar computational principles as the V1 [27,28]. The underlying is to generate multiple versions of ICA features at different levels, which when combined, will probably provide more stable predictions. Hence, we try to facilitate such a hierarchical ICA architecture at multiple levels on top of the first layer ICA outputs to capture the nonlinear statistical structures of the visual inputs that cannot be obtained by a single layer of linear ICA. The first layer of the ICA learning starts with an intelligent guess of the initial parameters, by feeding the model with a set of original input image patches **X**, so as to memorize the basic linear inherent nature reflected from the ICA directly. After the first layer of ICA filter, the sensory input set **X** will be represented by the ICA feature set **S** = (**s**_1_,…**s***_i_*,…,**s***_n_*). In order to better achieve the nonlinear statistical structures, the coordinate-wise nonlinear activation functions will be applied to transform each dimension of the first layer ICA output **s** to the second layer ICA input ***x***′ = *g*(|**s**|), so that the input of the next layer could satisfy the constraints imposed by the efficient encoding model. The statistical structures among dimensions of ***x***′ are then extracted by the next layer of linear ICA. The hierarchical model with more layers can be established to have a further understanding and improve the interpretation towards the early visual input from diverse perspectives. We will first take the above hierarchical ICA model into natural images to extract the generic common visual features, and then make use of the basis functions for the underwater images. One hierarchical ICA architecture is shown as Figure 4.

### 4.4. Algorithms

In our paper, the optimization criterion in the ICA is based on the negative entropy concept. One fast algorithm is taken here with the nonlinear activation function applied into the multilayer ICA architecture, which first selects an initial point and then replaces the original selection by updating the multiple independent components in the iterative process, and gradually achieves the convergence into a fixed point. The pseudo-codes of the steps are as follows:

**Algorithm 1**
1.Input all the image patches in the sample set **X** with the pre-whitening steps and initialize the basis function matrix **A**2.Calculate the mean vector ***m*** from the input image patches, 
$\text{m}=\frac{1}{n}\sum_{i=1}^{n}{\text{x}}_{i}$Construct the *d* × *n* mean matrix for the sample set **X**, **M** =(***m, m***,…,***m***)Apart from the mean matrix and get the centered sample matrix **X̅, X̅** = **X**−**M**3.Get a *d* × *d* covariance matrix, **C** = **X̅X̅**^T^ = **UΛU**^T^ with the diagonal matrix of the eigenvalues **Λ** = diag[*λ*_1_,…*λ_i_*,…,*λ_d_*] and the eigenmatrix **U** = [***u***_1_,…,***u****_i_*,…,***u****_k_*].Adjust **Λ** in a descending order and get the corresponding eigenvector ***u****_i_* by the arrangement of the eigenvalue *λ_i_*Apply the PCA projection and select the first *q*th eigenvectors to form the whitening matrix **V** in a *q* × *d* size, 
$\text{V}=\Lambda^{-\frac{1}{2}}{\text{U}}^{\text{T}}$, where **Λ** turns to be a *q* × *q* matrix and **U** a *q* × *d* matrix.4.Whiten the centered sample matrix **X̅** with **Z** = **VX̅** in a *q* × *n* size.5.Take the Fast-ICA algorithm and get the orthogonal matrix **B** = (***b***_1_,*…****b****_i_,…*,***b****_q_*) in a *q* × *q*size, **A** = **V**^−1^**B**, where the basis function **A** is a *d* × *q* matrix and the *i* th element ***b****_i_* in **B** is a *q*-dimensional vector. Compute the ICA feature coefficient matrix **S** = **B**^T^**Z** in a *q* × *n* size.6.Reconstruct the approximation of the original image sample set **X̃** with **X̃** = **AS**.7.Whiten again by Step 3 and get the *d* × *n* whitened matrix **S** by **S**= **V**^T^**S**.8.Discard the signs of the output **S** in this layer and take the nonlinear activation function g to form the input sample set **X**′ for the next layer, **X**′=g(|**S**|).9.Repeat the above process to achieve the recursive multiple layer ICA architecture


The steps of the fast fixed-point ICA algorithm are as follows.



**Algorithm 2**
1.Input the whitened sample matrix **Z** and set the maximum number of iteration for every orthogonal component as *T*.2.For *t* = 1:*T* For *i* = 1:*q*   Judge the condition, as the orthogonal matrix **B** needs to meet the condition formula **B**^T^**B** = **BB**^T^ = I, where I is the unit matrix, so each column vector ***b****_i_* in **B** is required to follow 
${\text{b}}_{i}^{T}{\text{b}}_{i}=1,i=1\ldots q$ and 
${\text{b}}_{i}^{T}{\text{b}}_{i}=0,i,j=1\ldots q,i\neq j$, *i.e.*, the norm ║***b****_i_* (*t*)║_2_ = 1 for each iteration *t*.   Take the following iteration formula 
${\text{b}}_{i}(t+1)=\text{E}\{\text{z}f\left({\text{b}}_{i}^{\text{T}}\left(t\right)\text{z}\right)\}-\text{E}\{f\prime \left({\text{b}}_{i}^{\text{T}}\left(t\right)\right)\}{\text{b}}_{i}(t)$, where E refers to the mathematical expectation and the activation function is 
$f(x)=\frac{e^{2x}-1}{e^{2x}+1}$.  Get the orthogonalization for the ingredients by 
${\text{b}}_{i}(t+1)-\sum_{j=1}^{j-1}<{\text{b}}_{i}(t+1),{\text{b}}_{j}>{\text{b}}_{j}$  Normalize ***b_i_*** by 
$\frac{{\text{b}}_{i}(t+1)}{{\left\|{\text{b}}_{i}(t+1)\right\|}_{2}}$  Continue the iteration until the convergence is achieved. EndEnd


The numerical results of the hierarchical ICA model with the above algorithms are shown layer by layer into the images in the next section.

## 5. Simulations and Result Analysis

**Figure 5.**
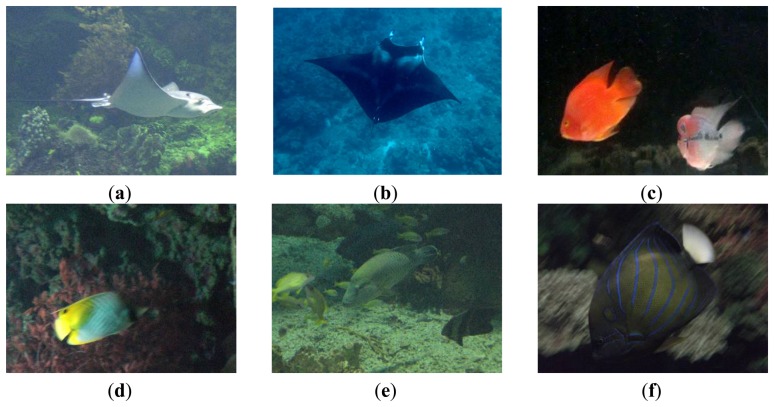
Sample underwater images.

In our simulation experiments, video recordings were collected by the C-Ranger AUV system with an average submergence depth of 2–3 meters. At each observation site, the environmental variables, including the ambient water temperature, current speed at the mooring location, the depth and the direction, as well as the survey-design variables, such as the AUV cruising speed and direction, the navigation and positioning, the altitude above the sea floor, AUV distance from the bottom, were recorded simultaneously. The LED lighting (typical lumens: 1,200 in the water, color temperature: 6000° K) was adopted for illumination when the natural sunlight are insufficient. Mixed with those images taken by the divers or underwater photographers together, the image database was formed to verify the performance of our proposed model. Figure 5 shows examples of the underwater images collected. All the simulation experiments have been run on the same x86_64 Windows machine with at least 4 GB of memory and 2+GHz processor. The execution environment is under MATLAB 7.0.

**Figure 6.**
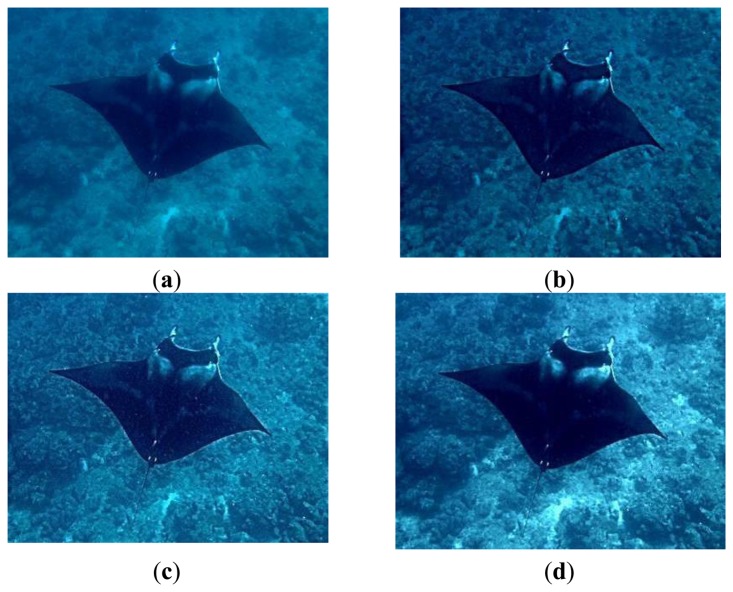
Image quality enhancement. (**a**) Original image. (**b**) Wavelet filtering. (**c**) Homomorphic filtering. (**d**) Image enhancement proposed.

Some preprocessing was then done to decrease noise or perform feature extraction in advance before formally ICA computation. The proposed underwater enhancement method was adopted here to get better image quality for the ocean investigations. Figure 6 lists the resulting enhancements for one example underwater image by the wavelet filtering, the homomorphic filtering, as well as the proposed method.

**Figure 7.**
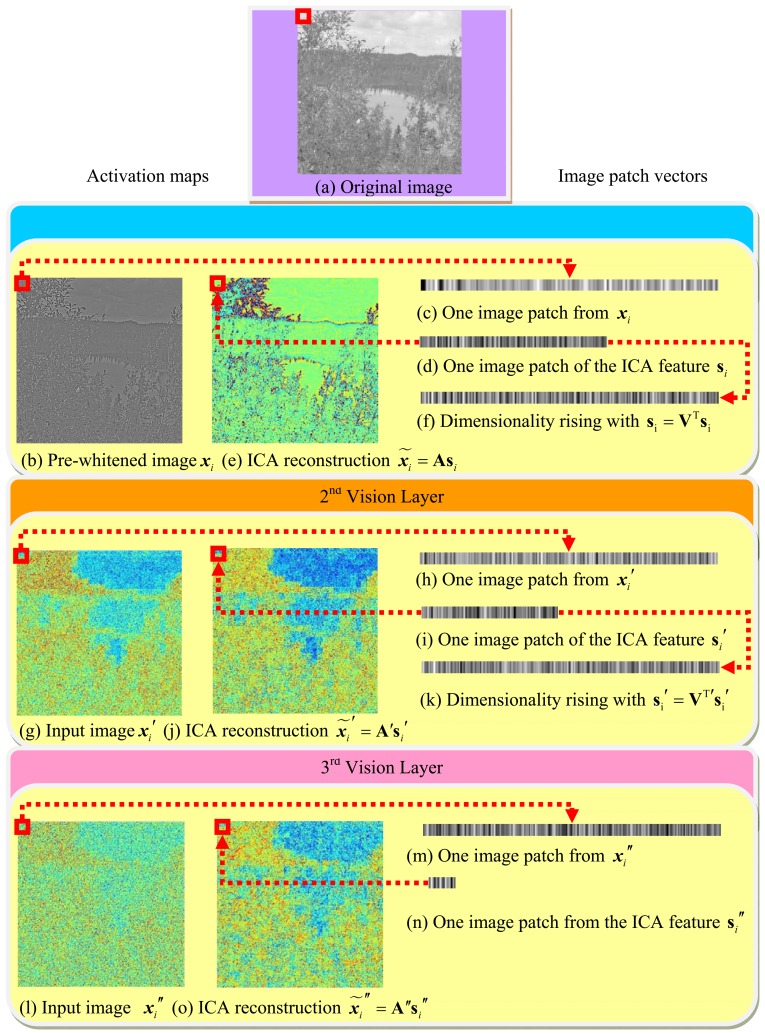
The hierarchical ICA computation process of one example image.

**Figure 8.**
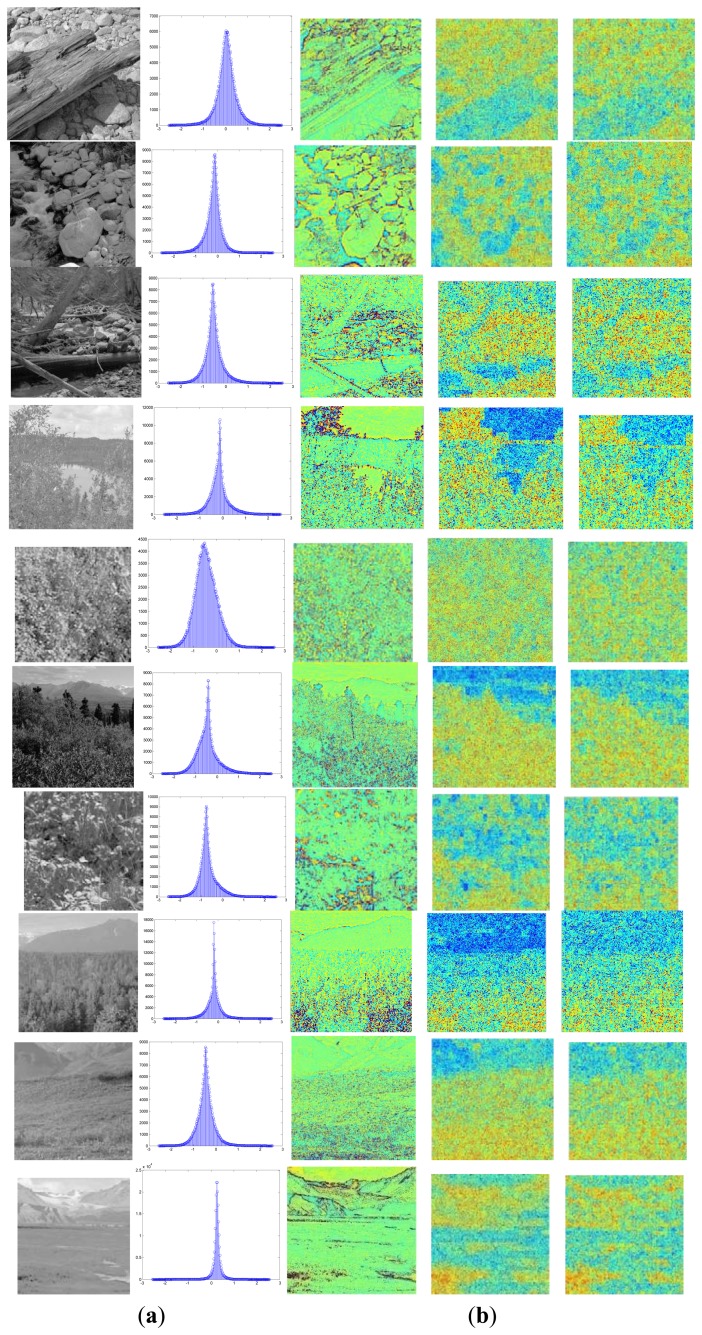
The activation maps for the natural images in each layer. (**a**) The original image and the distribution. (**b**) ICA responses in the 1st, 2nd, 3rd layer.

**Figure 9.**
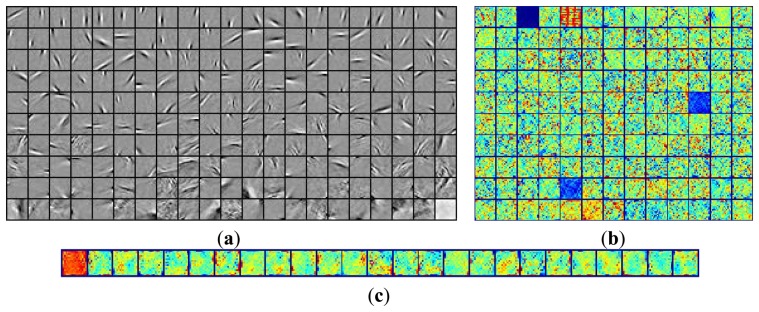
The basis functions. (**a**) Basis functions in the 1^st^ layer; (**b**) Basis functions in the 2^nd^ layer; (**c**) Basis functions in the 3^nd^ layer.

The common feature was first extracted from the natural images by the fast ICA algorithm. Figure 7 briefly summarizes the hierarchical ICA computation process for one example image. The way to extract the independent features, the activation maps, the dimensionality rising, the input image in each layer, as well as the corresponding image patches could be clearly seen here. Figure 8 shows the activation maps in each layer when the image patches are evenly distributed with *M* = 512, n = 10, *p* = 16, where the leftmost are the original natural images, the second column is the distribution of the pixel values in the whitened images, the third to the rightmost columns are respectively the corresponding activation maps from the first to the third layer. Table 1 lists the average running time of training one natural image in each layer for the hierarchical ICA architecture. Figure 9 illustrates the basis functions retrieved for the first layer, the second layer and the third layer respectively. We further tried to investigate what kind of statistical structures each layer is fond of. In particular, the first layer seems more activated by seemingly blank background, while another two layers seem to prefer the textures of the images.

**Figure 10.**
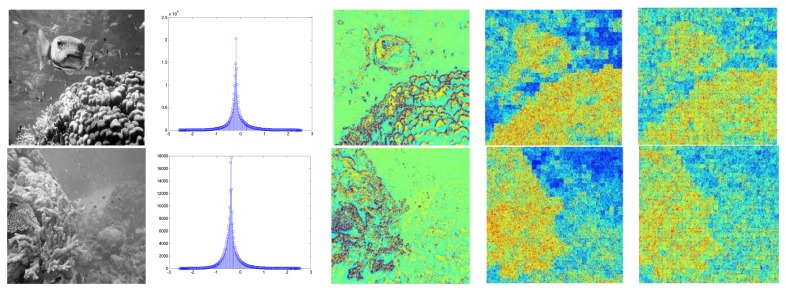

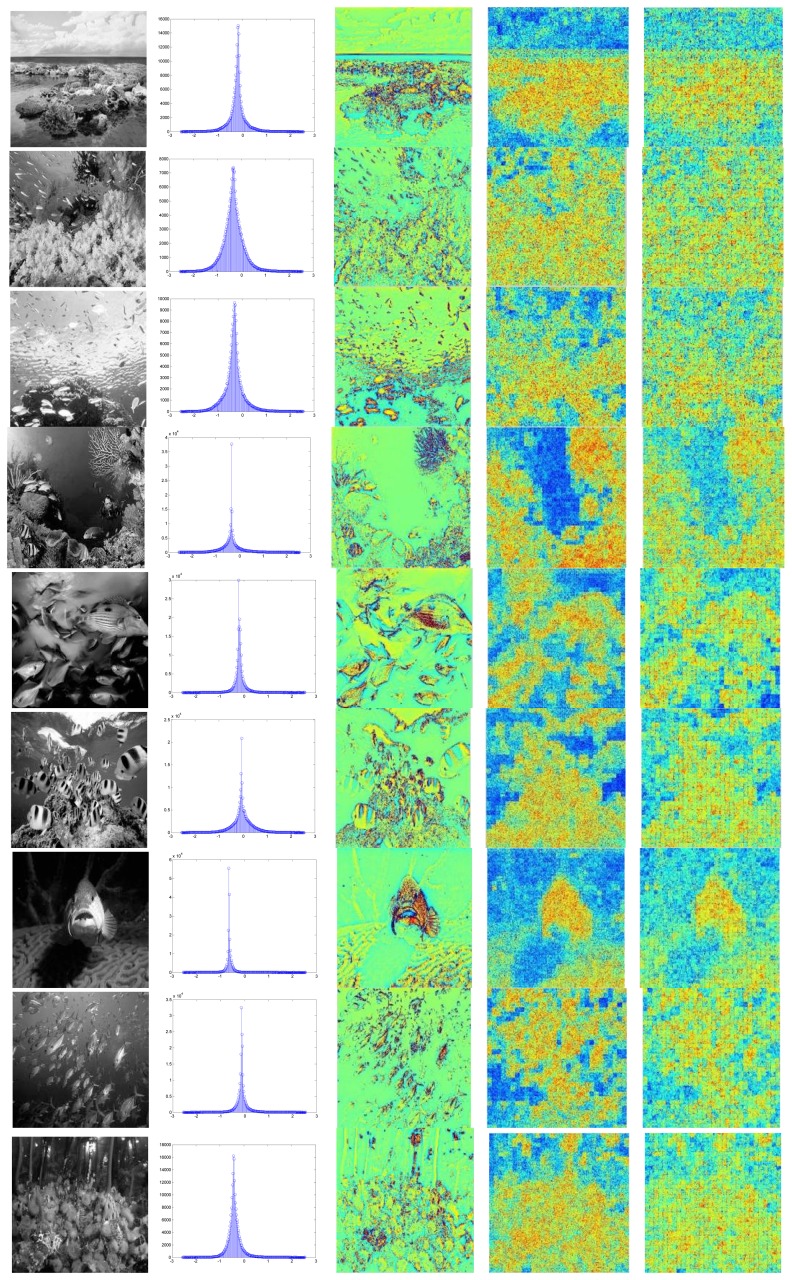

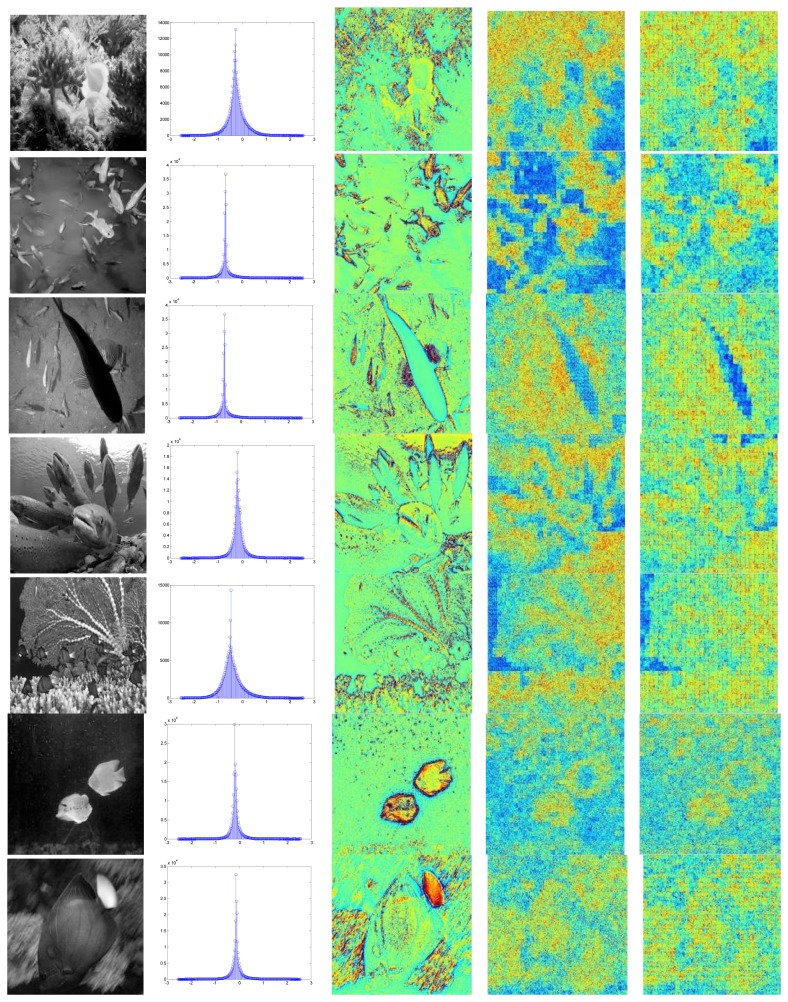
The activation maps for the underwater images in each layer.

**Figure 11.**
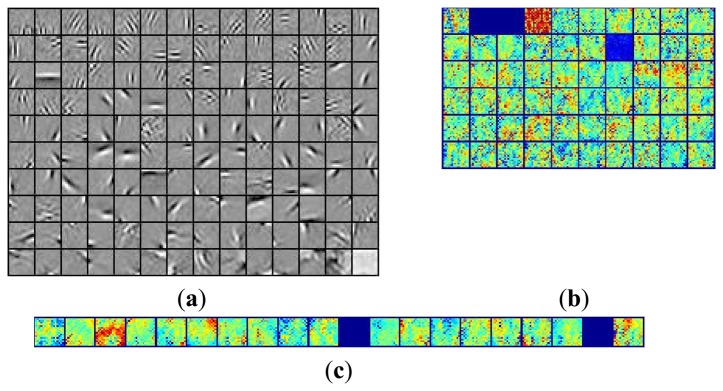
The basis functions. (**a**) Basis functions in the 1st layer; (**b**) Basis functions in the 2nd layer; (**c**) Basis functions in the 3nd layer.

**Figure 12.**
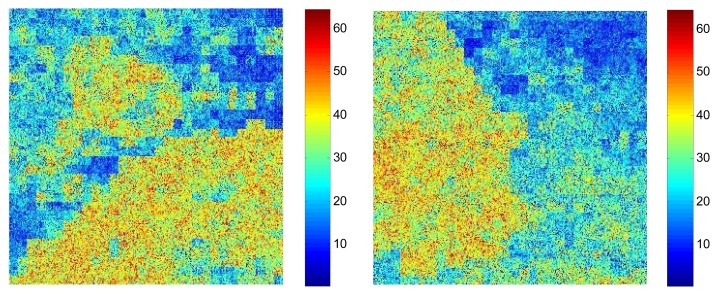
The color scale.

The underwater vision model was then initialized with the help of the basis functions extracted from the natural images by the fast ICA algorithm in each layer. Figure 10 shows the activation maps per layer when *M* = 512 and *p* = 16, where the leftmost are the original underwater gray-level images, the second column is the distribution of the pixel values in the whitened underwater images, the third to the rightmost columns are respectively the corresponding activation maps from the first to the third layer for the underwater images. Table 2 lists the average running time of testing one underwater image in each layer for the hierarchical ICA architecture. Figure 11 lists the basis functions retrieved directly from the underwater images for the first layer and second layer respectively. It is shown that the basis functions derived from the natural and underwater images are very similar to each other, so we can further make use of the common feature hypothesis for the other experiments in the ocean investigations. In this way, the proposed model could achieve good performance effectively in the very limited duration. Figure 12 shows the color scale of the activation maps in the Matlab 7.0 visualization, where the red color corresponds to the maximum value and the blue color refers to the minimum value.

From our underwater artificial vision model, it is shown that the ICA feature outputs in the first layer respond to the small local edges of the underwater objects, the second layer outputs are similar to the texture boundaries and shape contours, corresponding to the underwater image segmentation that could well distinguish the pure and complex background in the larger size of the textures, and the third layer outputs possibly have preferences for more detailed and complicated textures in the sea. Although we have no initial guess of what kind of statistical structures the second and third layer outputs might capture in the past, as is shown in the basis functions, some of the inputs in the second layer prefer strong activation within a certain orientation range but have no preference for locations, while others have a location preference but welcome activation of the first layer outputs of all frequencies and orientations, and some develop a picky appetite for both. The activation map of the third layer suggests that they might be tuned for the complicated textures. By accumulation, it will be more helpful to produce more clear ICA outputs in higher quality for each layer. The same procedure can be repeated and extended to more layers when we have better analysis and understanding in the ICA representation developed by the deeply embedded features in our model.

**Figure 13.**
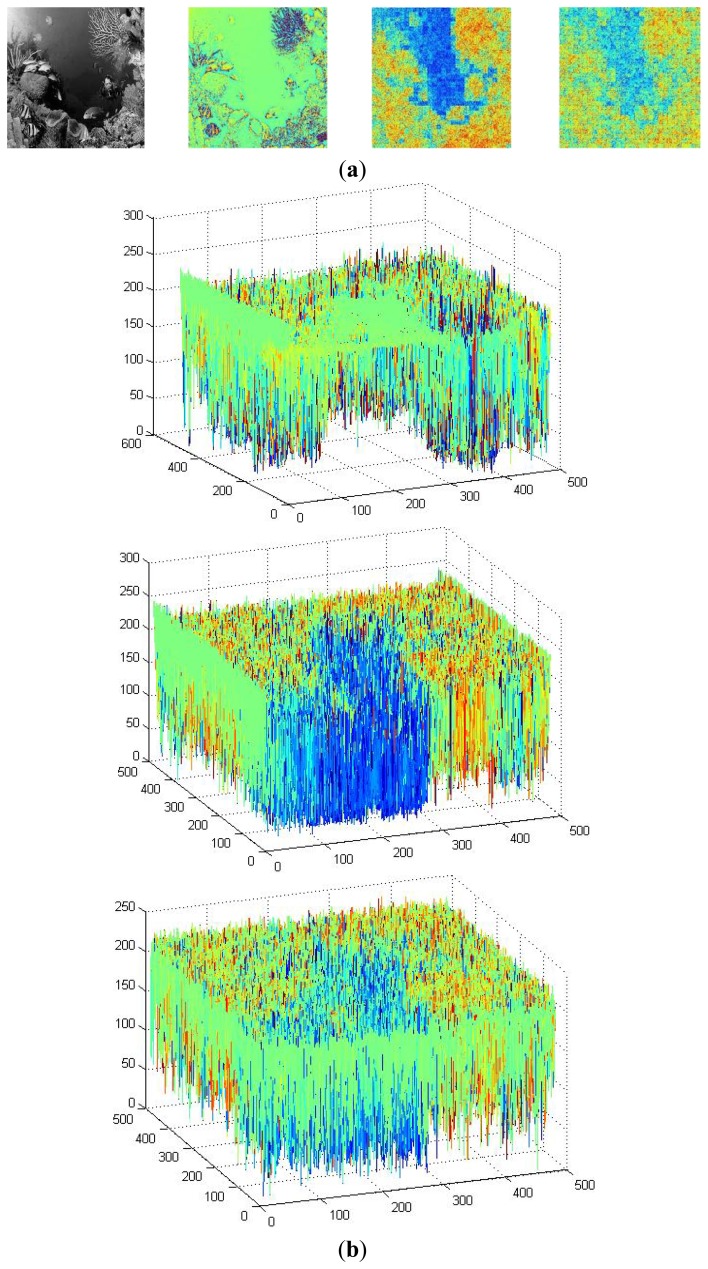

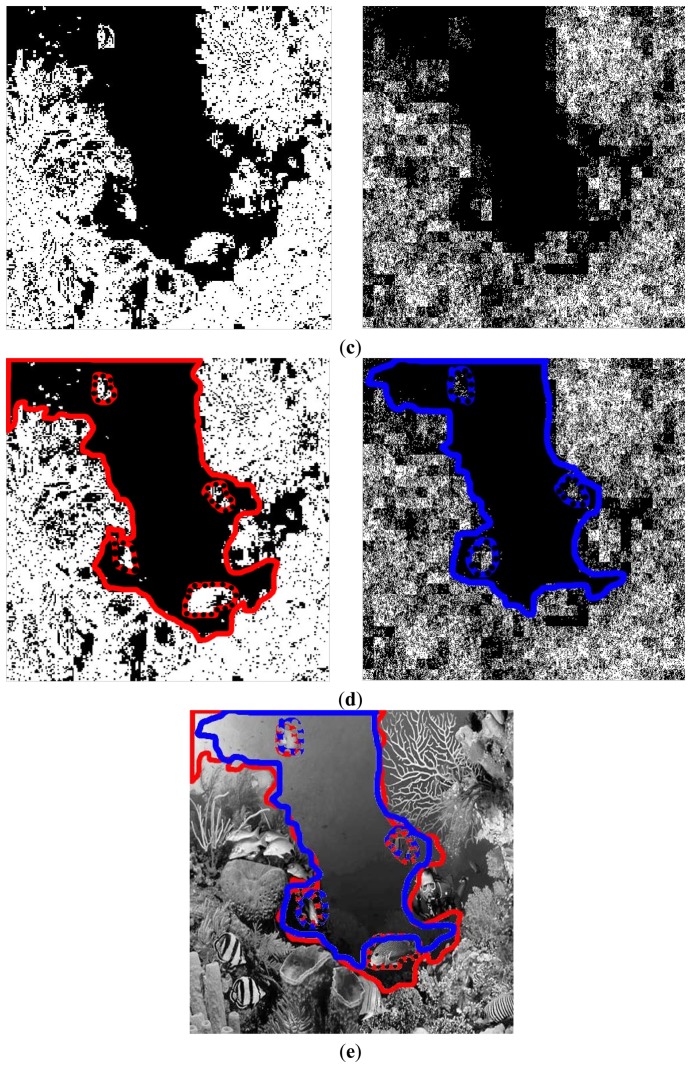
The feasibility of the navigation. (**a**) Original example image and the ICA responses; (**b**) 3D pixel map of the ICA outputs in the 1st, 2nd, 3rd layer; (**c**) Segmentation from the original image and the ICA response in the 2nd layer; (**d**) Traversable region recognition; (**e**) Navigation comparison.

Here our ICA architecture was also taken to explore the feasibility of implementing the potential underwater visual tasks. Figure 13 is one example of the navigation ability simulation for the ROV or AUV based system. It is shown from the 3D pixel map in Figure 13b that the difference of the ICA responses increase largely in the 2nd and 3rd layer, compared to the 1st layer, and we could benefit from the performance to evaluate the pure and large area region under the sea and hereby to provide the possible navigation information. Figure 13c lists the segmentation results respectively extracted from the original image by the region merge and split method as well as those from the ICA output with our proposed approach. Figure 13d shows the corresponding traversable region recognition, and the safe region is located within the solid line and outside the dotted line. Figure 13e makes a comparison with the navigation results in the example underwater image. In average, the proposed method could recognize 96.3% of the pure and large region in the sea in our database and provide us a good possibility for the navigation. Together with other sensors such as the sonar, we could have a more clear picture about the route planning for ocean investigations.

## 6. Conclusions

In this paper, we try to explore an early and elementary underwater artificial vision computational model. The basic ICA principle successfully used for the very first layers of visual information processing in the cerebral cortex has been extended and developed to explain the mechanism of the higher layers in a much complicated undersea environment for ocean investigations. The key idea of the model is to transform the high-order residual redundancy to linear dependency that can be easily exploited by the traditional ICA algorithm. The hierarchical ICA architecture has been set up with the same structure constructed at every level in the framework of our C-Ranger AUV system. As the redundancy reduction is dealt with progressively along the multiple layers, the nonlinear statistical structures of the underwater images in higher orders will be captured accordingly with a simplicity of generalization. The simulation results have shown the effectiveness and the consistence for the collected underwater images in our scheme. The first layer produces the Gabor-like basis functions, the second layer has some visual outputs with texture boundaries and shape contours, and the third layer shows interesting results that seem to have preferences for more complicated textures. The performance of the artificial vision model provide the possibility to help with the implementation of some potential underwater visual tasks, and an effective visualization understanding need to be further done in the future.

## Figures and Tables

**Table 1.** The average training time of the natural images.

**ICA Layer**	**1st Layer**	**2nd Layer**	**3rd Layer**
Running time (s)	131.3344	78.8672	29.7673

**Table 2.** The average test time of the underwater images.

**ICA Layer**	**1st Layer**	**2nd Layer**	**3rd Layer**
Running time (s)	18.6875	1.7204	2.1205
